# Aquatic versus Terrestrial Insects: Real or Presumed Differences in Population Dynamics?

**DOI:** 10.3390/insects9040157

**Published:** 2018-11-01

**Authors:** Jill Lancaster, Barbara J. Downes

**Affiliations:** School of Geography, University of Melbourne, Melbourne, VIC 3010, Australia; barbarad@unimelb.edu.au

**Keywords:** dispersal, drift, insect flight, herbivory, outbreaks, oviposition, North Atlantic Oscillation, parasites, population cycles, population regulation

## Abstract

The study of insect populations is dominated by research on terrestrial insects. Are aquatic insect populations different or are they just presumed to be different? We explore the evidence across several topics. (1) Populations of terrestrial herbivorous insects are constrained most often by enemies, whereas aquatic herbivorous insects are constrained more by food supplies, a real difference related to the different plants that dominate in each ecosystem. (2) Population outbreaks are presumed not to occur in aquatic insects. We report three examples of cyclical patterns; there may be more. (3) Aquatic insects, like terrestrial insects, show strong oviposition site selection even though they oviposit on surfaces that are not necessarily food for their larvae. A novel outcome is that density of oviposition habitat can determine larval densities. (4) Aquatic habitats are often largely 1-dimensional shapes and this is presumed to influence dispersal. In rivers, drift by insects is presumed to create downstream dispersal that has to be countered by upstream flight by adults. This idea has persisted for decades but supporting evidence is scarce. Few researchers are currently working on the dynamics of aquatic insect populations; there is scope for many more studies and potentially enlightening contrasts with terrestrial insects.

## 1. Introduction

The study of population dynamics is central to much ecological research. It is also a field with a history of controversy and debate, which ultimately galvanized a great deal of productive research. Although population dynamics has been a cornerstone of insect ecology for centuries, aquatic insects are seldom considered in this context even though much research on aquatic insects relates directly or indirectly to various key principles of population ecology. The general aim of this paper is to explore some similarities and differences, both real and presumed, between the ecologies of aquatic and terrestrial insects, and to identify areas where aquatic insects can make a significant contribution to a general understanding of population dynamics.

The study of population dynamics seeks to understand how and why population sizes differ over time and between locations, and how these changes are a function of demographic vital rates (births and deaths) and dispersal (immigration and emigration). Questions about population regulation focus on what prevents uncontrolled population growth and how populations grow from very low densities. First, a précis of the factors that collectively govern population dynamics will provide the overall context for the discussion that follows on particular factors affecting aquatic vs. terrestrial insects. In terms of reproduction, insects generally have *r*-selected life history strategies and produce very many eggs or offspring per female. The proportion of those eggs that survive to adulthood and are reproductively viable is critical in determining the potential for populations to grow. In terms of mortality, insects typically display Type III survivorship curves with high mortality in the early instars and the potential sources of mortality are diverse. Thus, knowing where females lay their eggs and how those choices influence the fate of their offspring is fundamental, as well as understanding the effects of environment, bottom-up and top-down controls on population size. Exchange of individuals between populations via dispersal is necessary for the long-term persistence of many species. Every emigrant is also a potential immigrant to another population, provided it is able to locate a suitable new habitat and also to reproduce there. Thus, dispersal must be successful in order to matter in a population context [1], i.e., individuals must arrive, survive and join the local population and, for adults, this also entails successful reproduction. Dispersal is therefore a key aspect of how populations are structured across landscapes. Thus, populations may comprise one large, patchy population distributed over relatively large geographic areas when successful dispersal is frequent. Alternative population structures include metapopulations, or variants thereof, where populations with mostly independent dynamics are connected by at least some dispersal, or isolated populations that are restricted to patches of habitat and where no dispersal takes place [2]. Discriminating between population structures can identify whether some populations require immigration to remain extant (so-called “sink” populations). Thus, dispersal frequency is a critical aspect and immigration may be as (or more) important than local births.

In this paper we explore some aspects of aquatic and terrestrial insect populations that are interesting because they suggest different processes predominate in the two ecosystems, or because there are incorrect perceptions in the literature about aquatic insects. This is not intended to be an encyclopaedic or exhaustive review, but rather to highlight some interesting aspects of insects and some recent developments in the study of aquatic insect populations. The overwhelming majority of aquatic insects inhabit freshwaters (or inland saline waters) with only a very few occurring in marine environments [3], and these freshwater species are the focus of this review. Taxonomically, there is some overlap in the orders of aquatic and terrestrial insects. Aquatic insects are usually split into fully and partially aquatic orders [3]. Fully aquatic orders (Ephemeroptera, Odonata, Plecoptera, Trichoptera, Megaloptera) have juvenile stages that are aquatic and adult stages that are terrestrial and virtually no representatives are classified as terrestrial insects. In the semi-aquatic orders (predominantly Hemiptera, Lepidoptera, Diptera, Neuroptera, Coleoptera), most species are fully terrestrial but a few have aquatic juveniles and some have air-breathing, aquatic adults. When illustrating various phenomena, we have selected the best examples, rather than attempt proportional representation of the various orders or of the inhabitants of running vs standing waters.

This review has four main parts. The first part (Section 2) considers density-independent and density-dependent factors that can determine maximum population size in aquatic insects, and whether they differ from their terrestrial counterparts. The second part (Section 3) discusses some of the differences—and misconceptions about differences—between terrestrial and aquatic insect populations, including differences in diet between terrestrial and aquatic herbivores, the apparent absence of population outbreaks in aquatic systems, oviposition behaviours, modes of dispersal, and the spatial arrangement of aquatic environments in the landscape. This is followed (Section 4) by rare examples of population cycles in aquatic insects, which suggest some over-lap with terrestrial insects in the underlying mechanisms. The remainder of the review presents and reviews some recent advances in two aspects of aquatic insect population ecology, which open new ideas and perspectives about aquatic systems and present some interesting contrasts with the dynamics of terrestrial insect populations. The first (Section 5.1) discusses oviposition site selection, including specialised oviposition on objects that are not necessarily resources for larvae but that appear to confer some benefit, and correlations between population size and oviposition site availability. The second (Section 5.2), focuses on the facts and myths about the dispersal of stream-dwelling insects.

## 2. Do Aquatic Insects Differ from Terrestrial Insects with Respect to Population Dynamics?

The factors that control maximum and minimum population size or density (e.g., mortality, competition, resource limitation) and their relationships with density have perhaps been the focus of most research into population regulation. In aquatic insects, much of the evidence regarding various density-independent and density-dependent processes comes from research on the aquatic juvenile life stages and, indeed, events during the juvenile stages often may be responsible for population regulation in insects generally. Other life stages of aquatic insects (eggs, pupae, adults) have received less attention in the context of population ecology. As we show below, many of the mechanisms influencing population sizes are broadly similar for aquatic and terrestrial insect populations. Examples for terrestrial species are common in most entomology textbooks; here we provide comparable examples for aquatic insects.

There is ample evidence of direct responses of aquatic insect populations to factors that operate in a largely density-independent manner, particularly changes in abiotic conditions during the juvenile life stages. Water chemistry or quality is an exemplar and naturally occurring variations in acidity or salinity, for example, are associated with variations in population size as well as the presence/absence of many species [4,5]. Anthropogenic impacts on water chemistry are also important, including organic and inorganic pollutants, and aquatic insects have long been important biomonitoring agents to assess water quality [6]. Impacts of water chemistry may be via direct physiological effects on individuals, growth and developmental rates [7] or somewhat indirect effects such as decreases in dissolved oxygen [8] or disruptions to species interactions [9,10,11]. Variations in water temperature between locations, as well as temporal fluctuations over diel or seasonal scales, typically affect population size via constraints on developmental rate, adult body size and fecundity [12,13,14], and these effects may be manifest as variations in phenology and voltinism [15,16]. The frequency and magnitude of physical disturbances, such as floods and droughts, can also reduce population sizes [17,18,19]. Mechanistically, these environmental impacts may involve density-independent mortality, but density-dependent mechanisms are also possible when, for example, the availability of disturbance refugia results in density-dependent mortality [20] and through changes in consumer–resource dynamics [21,22,23]. Of course, these interactions can be complex in detail and, for example, developmental trade-offs can also minimise disturbance-related reductions in fecundity and thus minimise impacts on population size [24,25].

Classic forms of density-dependent regulation of terrestrial insects are also manifested in some populations of aquatic insects and, again, most studies focus on juveniles. For example, resource or food limitation (or bottom-up control) have been demonstrated elegantly in river fertilization experiments [26] and multiple field experiments in which the densities and adult body sizes of detritivorous insects varied with densities of detritus [27,28,29]. Intra- and interspecific competition for resources does occur in aquatic insects [30,31,32], but direct links to long-term variations in population size have yet to be demonstrated convincingly. Foraging cannibalism, an extreme form of intraspecific competition, is common in juvenile aquatic insects although it is primarily opportunistic feeding rather than selection of conspecifics. Nevertheless, cannibalism can provide a mechanism for maintaining population size in resource-poor or suboptimal environments [33,34]. Regulation of aquatic insects by predators (top-down control) may reduce population sizes through direct consumption of larvae or eggs [35,36], but also via impacts on life history traits such as developmental time, adult body size and fecundity [37,38,39]. Finally, various parasites have been identified in diverse species of aquatic insects [40,41] and there is evidence that they can regulate populations [42,43] (see also Section 4.1).

In summary, there is clearly a great deal of similarity in the nature of the processes that can regulate populations of both aquatic and terrestrial insects. Most of this work on aquatic insects has focused on juveniles and complementary work on other life stages would be useful in order to determine whether regulation does indeed occur primarily in the juveniles. There are, however, also some differences in the characteristics of aquatic and terrestrial ecosystems that may lead to differences in the way various processes play out in populations or how frequently different mechanisms of regulation appear in aquatic vs. terrestrial populations.

## 3. Real and Presumed Differences in Aquatic and Terrestrial Systems

In this section, we consider whether differences between terrestrial and aquatic ecosystems or between the types of insects that inhabit them might create differences in the sizes of populations or the way they are regulated. We focus on broad differences that translate into effects on mortality, births or dispersal because these have direct effects upon population sizes. Exceptions to such differences inevitably exist, but our intention is to focus on general patterns rather than exceptions.

### 3.1. Diet and Differences between Terrestrial and Aquatic Herbivory

A defining characteristic of terrestrial insects is the predominance of phytophagous species, i.e., those species that eat some part of higher plants: [44,45]. Note that phytophagy does not typically include those species that eat pollen or nectar [46]. Most phytophagous insects are restricted to eight primarily terrestrial orders (Coleoptera, Diptera, Hemiptera, Hymenoptera, Lepidoptera, Orthoptera, Phasmida, Thysanoptera), and most are specialists in that they will only consume some plant species. Strong et al. [45] estimated that, for each phytophagous insect species, there is approximately one other terrestrial insect species that is a predator, parasitoid or that feeds on decaying organic matter. Specifically, Strong et al. [45] estimated that 46% of terrestrial insect species are phytophagous, and a similar estimate (45%) was offered by Weis and Berenbaum [47]. Other estimates, which use the total numbers of described insect species rather than just terrestrial species, endorse this proportion. Thus, Stork [48] estimated that a total of 850,000 to 1,000,000 insect species had been described thus far, and Bernays [46] estimated that at least 500,000 of them are phytophagous. To be an insect consuming some part of a higher plant comprises ≈25% of species of multicellular animals globally and is one of the most common ways of making a living on the planet [45,46]. It is perhaps not surprising then that the study of phytophagous insects dominates entomology and, therefore, our knowledge of insect populations. Courtney and Kibota [49] estimated that research on phytophagous insects constituted as much as ≈10% of all papers published in general journals like *Ecology* and far higher proportions of specialised journals (e.g., *Journal of Economic Entomology*).

In contrast, aquatic insects have broad diets that can make it difficult to categorise species as herbivores or predators per se. On the face of it, many aquatic genera appear to be predators [44], but trophic omnivory (feeding at multiple trophic levels) is characteristic of many aquatic insects [50,51]. Predatory species may be opportunistic or switch from predation to herbivory depending on food availability or life-cycle stage [52]. Alternatively, many aquatic taxa consume decaying organic matter but they also may switch to feeding on plants when available or scavenge animal carcasses [53,54]. One estimate suggests ≈20% of aquatic insects feed on living plant material sensu lat., but most of these scrape microalgae off surfaces and do not consume or specialise on particular species of macrophyte [44,55] (higher plants in aquatic environments are termed macrophytes). Aquatic insects that feed on macrophytes are predominately Coleoptera, Diptera, Lepidoptera and Orthoptera (i.e., predominantly terrestrial orders), and species with this feeding habit are more common in lakes and ponds (75% of species) than rivers, but they comprise a minority of aquatic insects overall [44].

These differences in diets of terrestrial and aquatic insects matter because they are likely to entail quite different constraints on insect densities and hence population dynamics. In terrestrial ecosystems, a minority of annual primary productivity is consumed by herbivores (7–18% [56]). As was recognised many decades ago [57], if most plant material goes uneaten, then the densities of terrestrial herbivores must be constrained well below the point where most terrestrial vegetation would be consumed. This interesting observation has spawned a long, on-going debate about what factors constrain the densities of terrestrial herbivores [56,57]. A recent review suggests that the numbers of terrestrial insect herbivores are limited by their predators and parasites and not by shortages of food [58]. In contrast, a higher proportion of annual primary production (predominantly algae) is consumed by herbivores in streams and lakes (20–51% [56]). Strong [59] hypothesized that the algae that dominates freshwater ecosystems is more vulnerable to grazing than higher plants (e.g., fewer secondary plant compounds or recalcitrant tissues) and that this vulnerability permits freshwater grazers to remove comparatively high amounts of algal biomass. In general, this hypothesis appears to be correct. Experimental removals of freshwater herbivores trigger increases in algal abundances, whereas the removal of terrestrial herbivores has only weak effects on their food plants [60] (which is consistent with limitation by enemies rather than by shortages of food, as discussed above). There are many caveats to this statement (this is a large and diverse literature), and it is also that true that many freshwater studies on food webs are of planktonic food webs [60], which rarely feature insects. Nevertheless, this outcome suggests that numbers of freshwater herbivorous insects may be more often limited by access to food than are their terrestrial counterparts.

Are these differences between populations of herbivorous aquatic and terrestrial insects real? One approach is to ask whether insects feeding on macrophytes in aquatic systems have strong effects on their resources (i.e., are similar to species feeding on algae) or are more similar to terrestrial insects. Wood et al. [61] showed that aquatic macrophytes suffer higher levels of herbivory when herbivores are at high densities (this differs from many terrestrial plants, as discussed above), but insects were the least effective herbivores (compared to mammals, birds, fish, crayfish, molluscs and echinoderms). Thus, the impact of terrestrial herbivores on plants is relatively weak, but the impact of aquatic insect consumers of macrophytes is even weaker. In natural environments, aquatic insects that feed on macrophytes rarely occur at densities high enough to do much damage, even for species that specialise on one or a few host plant species [62]. Note that biological control of nuisance macrophytes generally occurs when herbivorous aquatic insects (e.g., some weevils) are outside their normal distribution range and released from predation by natural enemies [62]. Thus, differences between terrestrial and aquatic herbivorous insects—which is in theory created by the dominance of flowering plants in the former and algae in the latter—does indeed carry over into those aquatic systems where insects are phytophagous.

### 3.2. Population Outbreaks

The sizes of some insect populations are relatively stable over long time periods, whereas others display extreme fluctuations, potentially over several orders of magnitude. Such fluctuations or outbreaks may be cyclical with a largely predictable periodicity, or may be irruptive with erratic or unpredictable outbreaks. This phenomenon of outbreaks has galvanized much research into insect population dynamics and produced an enormous literature. Studies of aquatic insect populations, however, are virtually absent from this literature. Note that the well-known and often spectacular mass emergence of some aquatic insects, including some mayflies [63], dragonflies and chironomids [64], is not a population outbreak but rather a life history trait, termed population synchronization, that occurs every generation [65].

Why are there so few studies of irruptive or cyclical populations of aquatic insects? The absence of examples may be because the characteristics of aquatic systems and aquatic insects are not conducive to outbreaks. First, there is a general consensus that outbreaks are most likely to occur in populations that are controlled or regulated primarily by one or only a few processes. Food webs in freshwater systems are often highly connected with many omnivorous species [51] and this trophic complexity could mitigate against outbreaks in populations of constituent species. Second, much of the research into population outbreaks has focused on phytophagous terrestrial insects because the impact on their food plants is often conspicuous, and well-studied species typically feed on plants that have economic value, such as forest insects [66,67,68]. If outbreaks in insect populations are peculiar to herbivory on angiosperms, then they are likely to be rare in freshwater systems simply because a minority of aquatic insect species consume macrophytes (Section 3.1). Third, populations of aquatic herbivorous insects, which feed mostly on algae, may be regulated in ways that are different to terrestrial herbivores, as noted in Section 3.1, and this may mitigate against outbreaks in aquatic herbivores.

Alternatively, population outbreaks simply may not have been noticed or documented for aquatic insects. Indeed, the long multigenerational time series (ideally ≥20 consecutive generations) that are required to describe population dynamics are scarce for any aquatic insect [69]. Theoretically, however, the factors and processes that underpin population outbreaks should apply to aquatic insects as much as any other *r*-selected organism. Fundamentally, rapid increases in population size require high reproductive rates and in this respect aquatic and terrestrial insects are similar, e.g., many aquatic insects produce hundreds to thousands of eggs per female and some can reproduce partheogenetically [70,71]. Similarly, the various processes that could bring about the collapse of very high population densities, e.g., classical forms of density-dependent regulation, do occur in aquatic insects (Section 2). Episodic irruptions in terrestrial insects often occur after environmental disturbances that disrupt trophic relationships [72]. Floods and droughts are perhaps the major forms of natural disturbances in freshwater systems and although much research has focused on the ability of insects to survive or recover from such events [73,74,75], disruptions to trophic interactions do occur [22,23] and theoretically could be associated with population irruptions. Cyclical population dynamics can arise when populations directly or indirectly track oscillations in the climate, such as the El Niño Southern Oscillation (ENSO) [76,77,78,79] and different, spatially distinct populations may cycle in synchrony with these large-scale climate patterns [80]. Terrestrial and aquatic systems are both affected by these climate oscillations so it is plausible that populations of some aquatic insects will cycle in concert. Population cycles also can be driven by exploiter–victim interactions, as in the classical Lotka-Volterra predator–prey models, and there is no obvious reason why this could not happen to aquatic insects.

Although some differences between aquatic and terrestrial systems are real, it is still theoretically possible for outbreaks to occur in populations of aquatic insects and this phenomenon may simply suffer from lack of documentation. Despite the scarcity of long-term data sets for aquatic insect populations, there are three examples of cyclical population behaviour and they appear to be underpinned by process similar to cyclical behaviour in some terrestrial insects. These examples will be discussed in Section 4.

### 3.3. Oviposition Site Selection

A pertinent source of differences between terrestrial and aquatic insects is in the strategies they use for oviposition and how these differences may affect population densities. Again, most information about insect oviposition comes from studies of phytophagous species, which lay their eggs in or around the plants that will be used for food by their larvae [81], or studies of predatory insects that prey on herbivores that oviposit on food plants [82]. Thus, there is a large literature on how females locate appropriate plant species, especially when suitable host plants are patchy or rare and surrounded by unsuitable vegetation. Much of this research focuses particularly on plant attributes (such as chemicals that can be detected by female insects) that influence successful oviposition or larval survival [83]. In contrast, although some aquatic insects lay their eggs on or within plants (particularly macrophytes), many do not attach eggs directly to any particular object or attach their eggs to inanimate objects such as rocks, wood or bark [84,85,86,87,88,89]. Notably, these species are often very selective about the characteristics or microenvironment of these objects, which distinguishes them from many terrestrial insects that oviposit on hard surfaces but are rather unselective (e.g., some praying mantis). Rocks and wood provide hard surfaces for attaching eggs but do not necessarily provide food for larvae of many aquatic insects (other than indirectly e.g., via biofilms).

Another interesting difference is that some aquatic insects broadcast fertilised eggs into water bodies from the air [3]. Broadcasting of eggs is not a strategy seen among terrestrial insects for probably self-evident reasons [90]. When dropped into flowing water, eggs may be dispersed by currents before eventually attaching individually to various objects via attachment devices on the eggs themselves [3]. Unfortunately these eggs are difficult to detect in natural river channels, so little is known of the distances they travel or their survivorship, but hatchlings may begin life dispersed well away from where eggs were originally oviposited.

As noted earlier, most adult aquatic insects are terrestrial, which means females that oviposit in or on the water must search for and detect suitable, aquatic oviposition sites from the air and/or by crawling or swimming underwater to inspect prospective sites. This provides an interesting contrast with host plant selection by terrestrial insects and could lead to systematic differences in the spatial arrangement of juvenile aquatic vs. terrestrial insects, with possible consequences for populations [91], which we explore in Section 5.1.

### 3.4. Sizes and Shapes of Habitat Patches and Connection to Dispersal

The distribution of patches of habitat across landscapes and the ability of organisms to disperse between them is key to understanding population structure and sizes of populations (see Section 1). Do these aspects differ between aquatic and terrestrial insects? There are some key characteristics of aquatic insects and environments that suggest this may be the case.

Freshwater environments comprise <1% of the Earth’s surface [92]. Thus, the total potential habitat size for aquatic species is a fraction of that potentially available to terrestrial species. However, the area is smaller still when we consider that not all parts of freshwater environments are suitable for insects. All parts of small, shallow ponds are usually occupied, but lakes are different. Insects cannot live in the plankton because virtually all species lack any means to control buoyancy [3] and lakes have little light—and potentially no food—in deep areas. The few exceptions include *Chaoborus* spp., a midge that can regulate buoyancy and a few species of chironomid that inhabit the profundal zone. Thus, insects inhabiting lakes are typically restricted to the narrow strips around lake margins, which means that the area of suitable habitat is much less than total lake area. From a topological perspective, therefore, the suitable habitat of lakes for most aquatic insects is closer to 1- than 2-dimensional. A similar effect occurs for large, deep rivers, whereas shallower, smaller channels can be fully occupied. Another key aspect is that, because most adult aquatic insects are terrestrial, the vegetation surrounding lakes and ponds or along the margins of rivers is also a critical habitat [93]. Rivers are particularly interesting in this respect because channels are corridors connected by both flow and suitable vegetation, as well as being organised into dendritic networks. In summary, the area of habitat suitable for aquatic insects overall is tiny, very patchily distributed (ponds), or presented as largely 1-dimensional shapes that may (rivers) or may not (lakes) be directly connected by water. These habitat shapes and distributions have excited interest because of the effect they might have on population sizes and dispersal across landscapes, and this may differ from the largely 2-dimensional habitat patches occupied by terrestrial insects.

Most adult aquatic insects can fly, but those of the fully aquatic orders (apart from the Odonata) are typically small, cryptic or inconspicuous, relatively poor flyers, and many are also short-lived and non-feeding [3,94]. These characteristics are likely to set firm limits on how far these species are able to disperse, particularly those species that occupy ponds and lakes where dispersal is accomplished only during the adult stage and may entail flight over considerable distances between water bodies. Rivers are different. Adults may disperse up and down river corridors without flying away from water. Additionally, the aquatic juvenile stages (and adults for those with a fully aquatic life cycle, e.g., some beetles) can potentially disperse considerable distances using the current (called drifting). How far individuals actually travel is largely unknown. Most insects cannot be individually tagged (although this is now changing) or observed, especially where dispersal occurs over many kilometres or through complex terrain, and laboratory observations do not necessarily capture how insects behave in the field; these problems beset research on terrestrial insects as well [95]. Moreover, as mentioned in the Introduction, simple observations of individuals in the act of dispersing are insufficient to document successful dispersal. As we will discuss in Section 5.2, these logistical difficulties mean that conclusive information about dispersal or population structure in river insects is actually scarce.

## 4. Population Irruptions and Cycles in Aquatic Insects

As mentioned earlier (Section 3.2), evidence of outbreaks in aquatic species is scarce, but examples do exist. Here, we describe three examples of cyclical population dynamics in aquatic insects and provide comparable or contrasting examples in terrestrial species. These examples include, (1) a caddisfly that is host to a parasite, (2) a chironomid with larvae that are generalist feeders (collector-gatherers), and (3) assemblages of stream-dwelling species with populations that cycle in association with climate oscillations. We have not provided an example of episodic irruptive behaviour analogous to outbreaks of Orthoptera under favourable environmental conditions [96] because we are unaware of any data demonstrating such behaviour in aquatic insects.

### 4.1. Example 1: Host–Parasite Population Cycles

**Figure 1:**
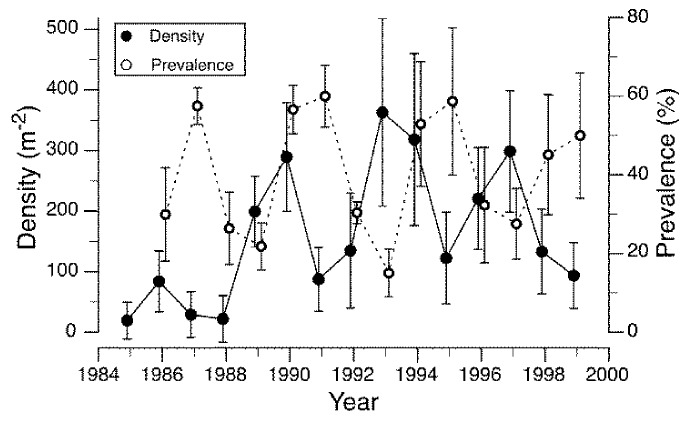
Larval densities (filled circles) of the univoltine caddisfly *Brachycentrus americanus* (mean ± 95% confidence interval (C.I.)) in 15 consecutive generations and the mean prevalence (open circles) of a microsporidium parasite, *Microsporidum* sp., in the larvae and pupae of *B. americanus* within each generation. Prevalence was calculated as the mean over all sampling dates in a generation, weighted by population density of the host on each date. Redrawn from [42].

Densities of a caddisfly, *Brachycentrus americanus*, and the prevalence of a microsporidium endoparasite exhibited cyclical dynamics with a periodicity of approximately four generations over a 15-year period, and the parasite appeared to be responsible for driving densities of the host caddisfly population (Figure 1) [42]. *Brachycentrus americanus* is a generalist filter-feeder and maximum population densities are likely to be food limited in many systems [97,98]. The microsporidium found in *B. americanus* attacks the fat body and other tissues of the host larval insect, which results in death of the host, usually before pupation. Analyses of these data revealed evidence of density-dependent growth of the caddisfly population, as well as density-dependent infection by the parasite with a lag time of one generation [42], as expected if a population is regulated by a parasite [99]. Analogous examples of cyclical population fluctuations driven by parasite-induced mortality include two terrestrial species pairs: the European corn borer (*Ostrinia nubilalis*) and its microsporidian parasite (*Nosema pyrausta*) [100], and the larch budworm (*Zeiraphera diniana*) and parasitic wasps (predominantly *Phytodietus griseanae*) [101]. Thus, it seems clear that cyclical population dynamics can be driven by host-killing parasites in at least one species of aquatic insect, and there may be others.

Cycles driven by host–parasite dynamics may be conspicuous where the parasite kills its host insect before the host reaches a reproductive stage (this example) or for sublethal infections where the parasite reduces host fecundity, although different mathematical models may be required to describe patterns underpinned by these two processes [102,103]. Reduced host fecundity via parasitic castration is typical for mermithid nematodes infecting aquatic insects [104,105]. The long-term population dynamics of these sublethal mechanisms have not yet been documented for aquatic species, but analogous sublethal infections can regulate some terrestrial insect populations [106]. Parasites of aquatic insects can also alter the behaviour of their hosts and make them more vulnerable to predation [107,108,109]; whether this process could influence population dynamics of aquatic insects requires further research, but cyclical behaviour is in theory possible [110].

### 4.2. Example 2: Consumer–Resource Population Cycles

**Figure 2:**
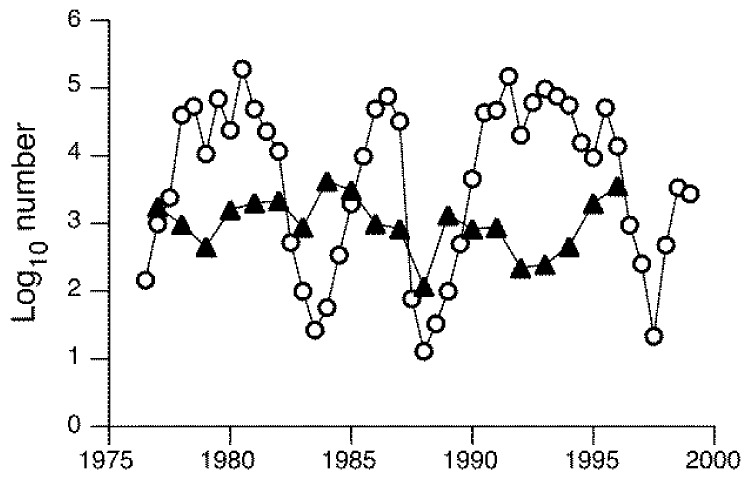
Window trap catches of adult chironomids, *Tanytarsus gracilentus* and *Orthocladius oblidens*, at Lake Mývatn, Iceland (trap site SN) between the years 1976 and 2000. *T. gracilentus* (open circles) is bivoltine with two, non-overlapping generations per year; *O. oblidens* (closed triangles) is univoltine. Redrawn from [111,112].

Multiple species of adult Chironomidae were sampled on the shoreline of Lake Mývatn, Iceland over 20 years [111]. A group of six species all exhibited cyclical oscillations in abundance over nearly six orders of magnitude and with an irregular periodicity of four to seven years [112,113], as illustrated by *Tanytarsus gracilentus* (Figure 2), which is a generalist feeder (diatoms, fine detritus) and the dominant species in this lake [114]. The absence of similar cyclical patterns in other species in the same lake, as illustrated by *Orthocladius oblidens* which is also a generalist (Figure 2), suggested that these fluctuations were not related to abiotic factors (e.g., weather) and, therefore, must have other explanations. Larvae in the group of cyclical species were associated with the profundal zone and feed largely on detritus and diatoms, whereas larvae of non-cyclic species were associated with macrophytes and the littoral zone, where they presumably exploited somewhat different resources. Using wing length as a surrogate for body size and hence resource abundance or quality for larvae, autoregressive models indicated that fluctuations in *T. gracilentus* numbers resulted from consumer–resource interactions rather than predator-prey interactions [112]. Exactly how and why their resources vary temporally is unclear. Further analyses suggested alternative dynamical states for populations of *T. gracilentus* in the absence of environmental variability [113]. Depending on initial midge densities, populations could fluctuate around either a fixed point or undergo high-amplitude cycles, thereby accounting for the irregular periodicity of fluctuations in abundance.

Examples of population cycles in terrestrial herbivores are numerous and most involve herbivory on angiosperms (Section 3.2). Despite the likelihood that constraints on populations differ for terrestrial and aquatic herbivorous insects (Section 3.1), cyclical patterns in population size do occur in at least one lake.

### 4.3. Example 3: Climate-Driven Population Cycles

Cyclical weather patterns have been associated with population outbreaks for various terrestrial insects, such as outbreaks of some Lepidoptera with the ENSO [76,77,78,79] and population fluctuations of some Hemiptera with the North Atlantic Oscillation (NAO) [115,116]. Many of these terrestrial species are herbivores and population cycles appear to be related to climate-driven variations in the quality and/or quantity of their food plants [117].

**Figure 3:**
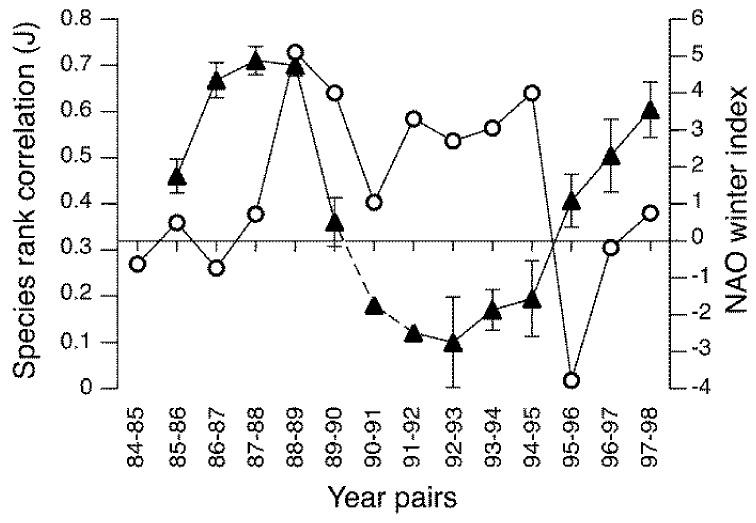
Year-to-year changes in the persistence of freshwater invertebrate communities (closed triangles, mean ± standard error (SE)) in eight streams of the Llyn Brianne catchments, Wales U.K., between 1985 and 1998. Persistence calculated as Spearman’s correlation (J) between the rank abundances of species in adjacent years. Dashed lines indicate missing data interpolated for 1991. Circles indicate the North Atlantic Oscillation (NAO) winter index. Redrawn from [121].

Similar examples of cyclical patterns in populations of aquatic insects are scarce and the available evidence suggests a different underlying mechanism. In Europe the NAO influences the water temperature of lakes, rivers and streams [118,119] and river discharge [120]. As shown in Figure 3, the NAO was also associated with fluctuations in the composition and relative abundance of macroinvertebrates (including insects) in some streams, with persistent assemblages during the negative phase (cold, dry winters) and unstable assemblages during the positive phase (mild, wet winters) [121,122]. Within these assemblages, significant correlations occurred between densities of some individual species and the NAO winter index, including negative relationships for *Nemurella picteti* (Plecoptera), *Elmis aena* (Coleoptera), *Hydropsyche siltalai* (Trichoptera) and *Paraleptophlebia submarginata* (Ephemeroptera), and a positive relationship for *Chloroperla tripunctata* (Plecoptera) [121]. These particular species encompass a diverse range of diets and feeding habits including algivores, detritivores, filter-feeders and predators, which suggests that population fluctuations may not be related to a changes in food resources, as suggested for some terrestrial insects (references above). Cyclical patterns of river discharge associated with the NAO were not a contributing factor to population cycles in headwater streams [121], whereas the effects of water temperature on the growth rate, final body size and fecundity of insects may play a role [123]. If correct, this suggests that climate-driven cycles in aquatic insect populations may be determined by direct, density-independent processes rather than via indirect alterations to food resources or trophic relationships. Of course, further examples are required to test this hypothesis.

Collectively, these three examples illustrate that cyclical fluctuations in population dynamics do occur in some aquatic insect species and the processes underlying these patterns appear to be analogous to those seen in terrestrial insects in some circumstance, e.g., host–parasite interactions, but not others, e.g., climate oscillations. However, the number of examples of population cycles from aquatic systems is very small and many more are required before generalisations can be made with confidence.

## 5. New Fields and Recent Advances

In this final section, we discuss two areas of research that are producing new insights into the population dynamics of aquatic insects: oviposition behaviours and dispersal. Thus, these research areas focus on rates that are essential to population dynamics (see Section 1) but that are seldom addressed in studies of aquatic insect populations. As a mea culpa, examples from our own research feature strongly in this section. This is not shameless self-promotion, but simply reflects the dearth of research in these areas and into the population dynamics of aquatic insects more generally.

### 5.1. Oviposition Behaviours and Aquatic Insect Populations

Oviposition behaviours, such as site selection, can have strong effects on the survival and fitness of offspring, as evidenced by an extensive literature on the preference–performance hypothesis for terrestrial insects. The spatial distribution of essential resources, such as oviposition sites, can also influence the spatial distribution of individuals within a population and such patchiness may have ramifications for population size [91,124,125]. Despite a wealth of research on terrestrial insects, only within the last ≈20 years has much research focused on understanding how oviposition behaviours affect populations of aquatic insects.

Apparently unselective oviposition behaviours, e.g., by broadcasting eggs above or at the water surface [3], may initially seem unlikely to influence population dynamics. Evidence suggests, however, that oviposition site selection for some species may occur at the larger scale of whole water bodies (e.g., ponds, rock pools, phytotelmata) based on the chemical composition of the water. Some volatile chemicals may become airborne and be detected by olfactory receptors on the antennae of flying females. Alternatively, females may also use contact chemoreceptors to test the water directly using sensilla on the ovipositor or distal abdominal segments [126] or other body parts, before deciding whether to oviposit. While chemical cues can be attractants [127], the majority of studies have demonstrated avoidance of water bodies inhabited by potential competitors or predators [128,129,130]. Such behaviour clearly could have profound implications for population size, particularly through impacts on the spatial arrangement of populations within landscapes.

Some aquatic insects oviposit very selectively on objects that do not necessarily provide resources for their larvae (Section 3.3). Furthermore, they often have strong preferences with respect to the characteristics or microenvironment of these sites [88,131,132], a degree of preference suggesting there may be some benefit to offspring. For example, caddisflies in the genus *Apsilochorema* (family Hydrobiosidae) lay jelly-covered masses of eggs attached to the underside of rocks, but only those rocks that emerge above the water surface in areas of slowly flowing water in rivers [88,132]. For this taxon, there is a clear advantage to selecting rocks in slow flows because the jelly on these egg masses is soft and egg masses laid in flows greater than ≈0.6 m/s are simply destroyed by hydraulic forces [133]. Other species in the same family have egg masses with firmer jelly that are oviposited selectively on emergent rocks in fast flows [88,132], but the hatching success of the predatory larvae is high in both fast and slow flows [133] so any potential advantages must be of a different kind. In contrast, some Odonata that oviposit endophytically and have predatory larvae (i.e., oviposition sites are not larval resources), prefer to oviposit in plant stems in fast flow where egg hatching success is higher than for eggs on stems in slow flows [134]. Of course, oviposition sites may be selected for the benefit of the female rather than her eggs or offspring. For example, adult females of some caddisflies in the family Hydropsychidae swim underwater to lay eggs on rocks in the slowly-flowing pools of rivers [85,135], yet their filter-feeding larvae typically occur in areas of fast flow. Swimming may be energetically efficient for adults in slow flows, but there are potential costs for early instar larvae having to move from the hatching site and locate suitable feeding locations.

**Figure 4:**
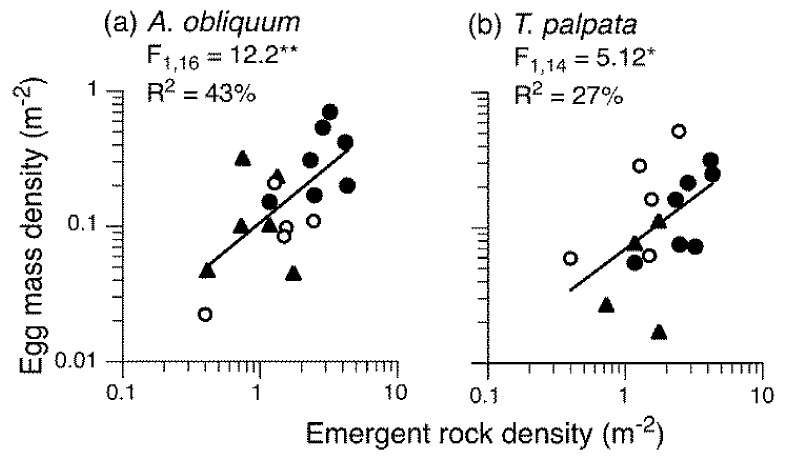
Relationships between the densities of egg masses and emergent rocks in different riffles for two species of caddisfly from different families, (**a**) *Apsilochorema obliquum* (Hydrobiosidae) and (**b**) *Tasimia palpata* (Tasimiidae). In each riffle, every emergent rock ≥5 cm (b-axis) and in ≥5 cm of water was examined for egg masses. Statistically significant relationships are indicated by regression lines and summary statistics. Data were log-transformed before analyses. Different symbols indicate riffles in different rivers, which were close together in Victoria, Australia. Riffles from multiple rivers were required to ensure a wide range of densities. Riffles with no egg masses were excluded from the analyses; preliminary statistical tests indicated that the nature of the relationships did not differ between rivers [144].

When species exploit specialised oviposition sites, the abundance of prospective sites may influence population size, and the spatial distribution of those sites may influence the spatial distribution of organisms within populations. Many species of aquatic insect oviposit eggs masses exclusively on emergent rocks in rivers, as in the example of *Apsilochorema* above. The cues females use to locate oviposition sites are poorly defined, but likely involve polarization vision [136] and potential disruptions to polarization patterns caused by broken or splashy water surfaces or mechanical detection of current speed [131,137,138]. The distribution of emergent rocks in rivers is patchy, with the highest densities in shallow riffles and much lower densities in the intervening deeper pools. The spatial arrangement of rocks within these patches as well as their environmental characteristics are important because females select oviposition sites in a spatially non-random manner, leading to aggregated distributions for some species and over-dispersion for others [132]. In terms of the abundance of emergent rocks, it is now well established that egg mass density is correlated with the density of emergent rocks in riffles or short river lengths for several species, including two species of baetid mayflies [84,87,139,140] and several species of caddisfly [141] (Figure 4). Similar positive correlations occur between whole streams that differ in the densities of potential oviposition sites [84]. Critically, the abundance of oviposition sites can be correlated not only with the abundance of egg masses but also densities of subsequent larval instars, e.g., two species of mayfly, *Baetis* spp. [141,142,143], and one species of caddisfly, *Rhyacophila dorsalis* [141]. This is compelling evidence that, even when oviposition sites provide no resources for larvae, the availability of oviposition sites can account for significant differences in the population sizes of some aquatic insects. In contrast, however, experimental manipulation of the abundance of egg masses and early instar of a megalopteran, *Sialis fuliginosa*, which oviposits on terrestrial vegetation, did not result in differences in the abundance of late instar larvae [36]. Thus, post-oviposition processes may cause these relationships to break down in some systems but not others.

### 5.2. Dispersal by Lotic Insects—More Myths than Facts?

Many lotic (stream-dwelling) insects drift and some do so in high numbers. The ubiquity of drift spawned a view of lotic insect populations that presumed downstream dispersal during the larval stage was frequent and would have to be offset by upstream dispersal by adults otherwise it would be impossible for insects to persist in river environments [145]. A corollary of this view is the so-called “drift paradox” because, despite drift, upper stream reaches are not defaunated over the long term. The apparent ubiquity of insects in the drift has also led many researchers to assume, often implicitly, that frequent dispersal links most suitable habitat within catchments, which therefore means that aquatic species have large, patchy populations [146].

In reality, remarkably few studies have tested whether drift is a significant source of successful dispersal or population loss. Instead, most drift studies simply document the densities of animals in the drift at points in time or space [147,148], even in relatively modern literature [149]. Some studies have measured drift distances [150,151,152] but over small temporal and spatial scales, so it is unclear whether these movements have population consequences. Only a handful of studies have quantified either drift distances or drift success over scales that might reflect population processes, and they present disparate results. Thus the valuable, relatively large-scale study of an Arctic river by Hershey et al. [153] showed that larvae of a mayfly, *Baetis*, drifted successfully ≈2 km downstream (and adults flew similar distances upstream). In contrast, in the U.K. Lancaster et al. [139] found that *Baetis rhodani* exiting riffles dropped out of the drift in pools and rarely reached the next riffle. These data were consistent with other evidence (Section 5.1) suggesting that drift dispersal did not greatly redistribute *Baetis* mayflies, a result consistent with that of Encalada and Peckarsky’s [143] study of *Baetis bicaudatus* in the U.S.A. Likewise, Brooks et al. [154] documented that pools can act as natural barriers to drift of multiple species, an effect that was magnified by an unnaturally large pool created by a weir [155]. In contrast, drift was prevalent in driving benthic density increases during a 12-month landscape-scale experiment that boosted resources of food and living space at sites distributed along a stream gradient [156]. Drift was associated with benthic densities in ≈60% of common taxa, and some upstream species moved downstream by up to 20 km by using manipulation sites as stepping-stones [157]. Not all drifters successfully colonised experimental sites, however, and some of the unsuccessful species were closely related taxonomically to species that were successful. Strong differences between related taxa were also documented by Downes and Lancaster [158] who found larval densities of the hydropsychid caddisfly *Smicrophylax* sp. AV2 were driven by successful drift but densities of the co-occurring and confamilial *Asmicridea* sp. AV1 were not. Such results are a warning that drift propensity is not a family- or genus-level trait. In the context of population ecology, it is important to demonstrate whether drift is dynamically important. Classic studies on an amphipod (not an insect), with a high propensity to drift, demonstrated that losses via the drift were trivial compared to population production [159]. Finally, upstream movement of juvenile aquatic insects can also be significant [160] and possibly offset the effects of drift dispersal, but studies have been few and some suffer from methodological shortcomings [161]. In short, the evidence that downstream drift is a dynamically important characteristic of lotic insect populations is both thin and unconvincing.

Likewise, the evidence that winged adults fly predominantly upstream for most species is also unconvincing. There has been no experimental test of the hypothesized colonisation cycle and simulation models suggest that upstream-biased dispersal of adults is not sufficient or necessary for population persistence [162,163]. Some studies have found upstream flight (e.g., the aforementioned study by Hershey et al. [153]) but, among the relatively few studies published, some have documented roughly equal numbers flying up- and downstream or that adults dispersed laterally away from river channels [164,165]. Thus, some aquatic species may disperse predominantly upstream, but this is not a characteristic feature of all aquatic insects [3]. Studies using radioactive or stable isotopes have shown that some adult caddisflies, mayflies and blackflies are capable of travelling multiple kilometres, especially where wind assists flight [164], but other studies found adults travelled no more than a few metres from the water [166], which suggests a wide range in propensity and ability to disperse among both species and individuals [3]. Like drift studies, most research on adult movement lacks evidence to demonstrate how often adult dispersal is successful (i.e., results in recruitment). Thus, adults may disperse to water bodies but fail to reproduce [167,168]. Such itinerancy, as it is called, is under-studied but could be common [1].

Clearly, both drifting and adult flight (followed by recruitment) may be a source of immigrants and emigrants for populations, but which route is more important? A rich source of information about this comes from work on population genetics. Dispersal frequencies that affect genetic connectivity do not quantify dispersal frequencies that affect population densities or growth rates [169], but genetic variation can identify the scales over which dispersal does not occur and suggest barriers to dispersal. Thus, if dispersal is mainly carried out via the drift (adults do not disperse much), then there should be strong genetic variation between populations in different streams within the same catchment but little variation among populations along the same stream (the stream hierarchy model, SHM). Alternatively, if adult flight followed by recruitment is the major dispersal mechanism, then this creates panmixia at small scales regardless of catchment boundaries [170,171]. The majority of results on aquatic insects are inconsistent with the SHM and show that adult flight creates significant genetic structure, typically at scales of ≈100 km except for species known to be exceptionally poor fliers or constrained by geographic barriers, such as deep canyons [170,171]. Interestingly, some species showed strong genetic variation at very small scales: among stream pools along the same channel [172,173,174]. The simplest explanation is that larvae within pools arose from only a handful of egg masses (the patchy recruitment hypothesis), which, again, emphasizes the point that recruitment is the yardstick of successful adult dispersal.

In summary, dispersal by stream insects is still greatly under-studied. It is likely that for many species both drift and adult flight play roles in setting population structure [157], but there is little evidence that populations are characterised by frequent drift dispersal downstream followed by adult flight upstream, nor is this necessary for population persistence [162,163]. Even so, modelling studies continue to be published that assert that downstream drift is pervasive among stream insects, and results in long-distance dispersal that must be countered by upstream flight by adults [175,176,177] in a seeming triumph of myth over evidence. It is possible that, despite the dendritic network of rivers, adults commonly fly across catchment boundaries and are not constrained by the dendritic network that forms the basis of so many models. If so, the spatial structure of stream insect populations remains largely speculative, and whether they differ from terrestrial insects remains an open question.

## 6. Conclusions

From this review it appears that aquatic and terrestrial insect population dynamics are clearly different in some respects, share similarities in others, and some presumed differences lack supporting evidence. The basic mechanisms underlying changes in population size, e.g., factors that act typically on larvae in density-independent and density-dependent ways, appear similar in both ecosystems. Clear differences lie in the way herbivorous insects relate to their food plants in the two ecosystems (i.e., higher plants vs. algae). Accordingly, populations may be regulated or constrained in different ways and this could lead to different dynamics. Although episodic, irruptions in populations of aquatic insects have not been documented, cyclical patterns in population size occur for populations inhabiting both ecosystems. The mechanisms underlying these cycles may be similar in some situations (e.g., parasite–host relationships), but different in others (e.g., populations tracking climate fluctuations; herbivore–resource dynamics), although examples are few for aquatic insects. Behaviours around oviposition site selection of some aquatic insects suggest that preference–performance relationships may be common in both aquatic and terrestrial species, even though oviposition sites may not be resources for aquatic larvae. However, some specialised oviposition strategies (e.g., laying eggs exclusively on emergent rocks) can strongly influence the spatial arrangement of individuals within populations, and can lead to strong constraints on population size through the availability of oviposition sites. Examples of comparable constraints on terrestrial insect populations are scarce. Finally, within landscapes, the shape and spatial arrangement of aquatic environments that insects can inhabit are very different from terrestrial systems, and this will have strong implications for the role of dispersal in aquatic vs. terrestrial insect populations. Nevertheless, long-held presumptions about the magnitude and effect of drift dispersal on populations of stream-dwelling insects are not supported by evidence. A major paradox is that many researchers persist in claiming there is a “drift paradox”, despite the absence of evidence.

Collectively, these conclusions suggest that our understanding of insect population dynamics can be enriched by further research on aquatic insects because they display some behaviours and population patterns that contrast with terrestrial insects. For example, it is possible to identify aquatic insect egg masses to the species level in the field [178], and fairly easy to quantify egg mass densities quickly and reliably over relatively long river lengths. Hatching success can also be monitored in the field (references above). Thus, there is considerable scope for research on whether insects in general have strong preferences for oviposition sites (e.g., water velocity, rock size), and, if so, how these preferences affect the density and distribution of egg masses and hatching success of larvae. It is also feasible to conduct experiments to test the preference–performance hypothesis and hence determine whether female aquatic and terrestrial insects behave in similar ways. Over larger scales, surveys of rivers offering different densities of emergent rocks can be used to test whether insect populations are constrained by female access to oviposition habitat, as described above. Moreover, variability in discharge in rivers, which can either drown or strand emergent rocks, means that temporal variability in oviposition habitat is also likely and could be another key source of variation in population numbers [87].

Furthermore, the shape of aquatic environments with juveniles restricted to water means that many are well-suited to studies of dispersal. Some ponds and lakes are only seasonally wet, i.e., ephemeral. The re-filling of dry ponds and lakes is followed by the dispersal of adult insects, which means hypotheses about dispersal rates (and their implications for populations) can be tested. Rivers are particularly suited to studies of dispersal. The downstream directionality of drift means that the rate of immigration into locations—and emigration out of them—can be reliably quantified, and hypotheses of whether dispersal drives benthic densities of larvae can be tested (references given above). Adults can be trapped and their numbers correlated with the densities of egg masses to test the role of adult dispersal and recruitment in driving larval numbers.

Much research in freshwater systems focuses strongly on community-level phenomena and the use of insect assemblages to assess ecosystem health; this means that relatively few researchers examine population-level phenomena. There is scope for much more insightful research on aquatic insects and appropriate methods have already been developed. There are, we suspect, a great number of interesting patterns waiting to be uncovered that could lead to some general understandings of population regulation in insects.
